# Supplementary material for: Are carbon emissions trading and green financial instruments synergistic? -Comprehensive quantitative research based on content analysis
Source: PLoS One. 2024 Mar 7;19(3):e0298601. doi: 10.1371/journal.pone.0298601 (PMC10919601; doi:10.1371/journal.pone.0298601)
Supplement: S1 Data — (ZIP) [file pone.0298601.s001.zip › Data/地级市Z_F工作报告.zip!/╣≤╓▌/┴∙┼╠╦«╩╨/└┤╫╘╣1⁄2╓┌║┼í╛┬φ┐╦╩2╛▌═°í┐_2007.docx]

各位代表：

　　现在，我代表市人民政府，向大会作工作报告，请予审议。并请各位政协委员和列席的同志提出宝贵意见。

第五届市人民政府工作回顾

　　五年来，在市委的正确领导下，在市人大、市政协的监督、支持和帮助下，我们紧紧依靠全市各族人民，以邓小平理论和“三个代表”重要思想为指导，全面贯彻落实科学发展观和构建社会主义和谐社会的重大战略思想，抢抓机遇，开拓进取，真抓实干，全面和超额完成了“十五”计划，实现了“十一五”良好开局。

　　过去的五年，是经济实力显著增强的五年。预计2006年生产总值达256.05亿元，比2001年翻了一番多，年均增长15.9%；财政总收入和地方财政收入分别达37.2亿元和16.8亿元，比2001年净增了28.24亿元和12.04亿元，年均分别增长33.01%和28.49%；累计完成固定资产投资434亿元，是前五年的3.2倍；工业增加值达135.37亿元，年均增长19.4%；金融机构存贷款余额分别达227亿元和170亿元，是2001年末的2.6倍和2.3倍。

　　过去的五年，是农村经济稳步发展的五年。加大了对“三农”的投入，2006年市级财政投入支农资金达8655万元。落实了粮食直补、良种补贴、农机具补贴等支农惠农政策。实现了农业税全免。农村基础设施建设力度加大，实施农村饮水安全工程，解决了44万人饮水安全问题；完成了两期农村电网改造，农户同网同价面达78%。建成通村公路3700公里，实现了村村通公路；建成沼气池5.5万口；完成了30万亩以中低产田土改造和坡改梯为主要内容的基本农田建设。粮食综合生产能力逐年提升，全市粮食总产量跃上80万吨台阶。农民培训力度加大，累计培训农民240.3万人次，转移农村富余劳动力26.7万人次。新阶段扶贫开发力度加大，净减少贫困人口4.7万人。实施易地扶贫搬迁、地质灾害搬迁、农村贫困户住房改造等工程，共完成15852户住房建设。启动了社会主义新农村建设。

　　过去的五年，是经济结构调整成效显著的五年。第一产业增加值由13.46亿元增加到21.3亿元，第二产业增加值由48.25亿元增加到149.8亿元，第三产业增加值由36.26亿元增加到84.95亿元，第一、二、三产业增加值比例由2001年的13.7∶49.2∶37.1调整为2006年的8.3∶58.5∶33.2。农业产业结构调整力度加大，农业产业化进程加快，畜牧业产值占农业总产值的比重由28%升至37%，马铃薯产业工程启动实施。“西电东送”工程建设实现重大突破，盘南电厂、发耳电厂、野马寨电厂、响水煤矿、发耳煤矿等一批重大项目相继开工建设并陆续投入运营，煤炭、电力、钢铁、建材等产业的支柱地位进一步巩固。主要工业产品产量大幅度增加，煤炭产量达4583万吨，是2001年的3倍；发电量达169.1亿度，是2001年的2.8倍；钢材产量达220万吨，是2001年的1.77倍；水泥产量达221.3万吨，是2001年的1.6倍。煤化工、高载能等产业进入了新的发展阶段，老鹰山煤基气化替代燃料项目一期工程已经核准，双牌铝业公司电解铝技改工程和铝加工项目建设进展顺利。交通运输、金融保险、餐饮娱乐、商贸流通、邮政通信、房地产、旅游等服务业加快发展。

　　过去的五年，是基础条件明显改善的五年。交通建设取得突破性进展，铁路“三线一站”投入运营，形成了西南地区重要的铁路交通枢纽；公路建设力度加大，镇胜高速公路境内段开工建设，水黄高等级公路建成通车，建设了一批二级公路、三四级经济路和通乡油路，实现了到省城通高等级公路、县区和绝大多数乡镇通油路。水利建设力度加大，玉舍供水工程、市中心区防洪一期工程建成投入使用；完成了9个病险水库的除险加固；建成“三小”水利工程344处、烟水配套工程1.53万亩，新增、改善和恢复灌溉面积23.74万亩；治理水土流失面积491平方公里。

　　过去的五年，是人口资源环境工作成效较好的五年。人口计生工作取得较好成绩，符合政策生育率从2001年的70.87%升至2006年的96.1%，人口自然增长率从13.23‰降至6.13‰。资源综合利用得到加强，节能降耗积极推进，淘汰了一批落后生产能力。环境保护力度加大，累计投入环保治理资金8亿多元，全市70%以上工业污染源的主要污染物达标排放，实现了增产不增污，环境质量稳中有升。以退耕还林为重点的生态环境建设成效明显，累计完成营造林175万亩，森林覆盖率由21.7%升至26%，生态环境恶化的趋势得到有效遏制。

　　过去的五年，是城市面貌焕然一新的五年。完成了第三轮城市规划修编。城市主干道建设和改造力度加大，城市总体骨架基本形成。供水二期、排污收集系统、城市燃气等综合管网工程建设步伐加快。垃圾处理、污水处理设施投入运行。人民广场、凤池园等公共场所建成并开放使用。城市绿化工作得到加强，环城林带建设取得较大进展。双水、凤凰、荷城、德坞新区建设起点高、速度快、效果好，市中心区建成区面积达36平方公里。市中心区城市管理体制基本理顺，城市管理力度加大，市容市貌明显改善。着力打造凉都品牌，成功举办了三届“凉都消夏文化节”，城市知名度和影响力逐步提升。县城和重点小城镇建设步伐加快。全市城镇化水平达27.2%。

　　过去的五年，是改革开放力度不断加大的五年。完成了一批国有企业的改革改制，盘活存量资产6亿多元。深入开展地企共建，实现了地企共赢。非公有制经济快速发展。农村税费改革全面完成。财政体制改革深入推进。农村信用社改革取得阶段性成效，城市信用社加快发展。积极推进政府管理创新，建立了市政府政务服务中心，行政审批效率得到提高。电子政务建设稳步推进。投融资、粮食流通、教育、文化、卫生等体制改革协调推进。投资软环境建设取得较好成效，对外开放进一步扩大，招商引资实际到位资金累计达132亿元。进出口贸易呈现良好发展势头。

　　过去的五年，是社会事业全面进步的五年。教育事业发展实现重大突破，共投入义务教育资金16亿元，城乡办学条件明显改善，全面完成了“两基”攻坚目标。组建了六盘水师范高等专科学校实验中学、六盘水职业技术学院、贵州大学六盘水能源矿业学院，普通高中教育、中等职业技术教育、高等教育加快发展。卫生事业稳步推进，医疗卫生服务体系日趋完善。文化、体育、科技、广播电视、新闻出版、气象、档案、老龄、残疾人、红十字、慈善等各项社会事业全面进步。

　　过去的五年，是民生问题得到较好解决的五年。新型农村合作医疗试点工作顺利推进，3个试点县参合率达74%，累计为农民报销医疗费4827万元。就业再就业工作连续四年超额完成任务，劳动保障工作获国务院表彰。新增城镇就业7.17万人，城镇登记失业率控制在4.4%以内。社会保障体系进一步完善，城镇低保对象实现了应保尽保，累计发放城市低保金2.42亿元；城镇职工基本养老保险、医疗保险、失业保险、工伤保险等制度逐步完善。6.2万农村特困群众纳入了长期定期救助范围。市级财政累计投入3203万元资金用于农村贫困家庭和城镇低保家庭民用煤补助。安全生产形势逐年向好，2006年安全生产指标控制在省下达的范围内并实现“双降”，煤炭百万吨死亡率为2.12，接近全国平均水平。人民生活水平较快提升，2006年城镇居民人均可支配收入达9201元，比2001年增加4055元，年均增长12.3%；农民人均纯收入达2034元，比2001年增加674元，年均增长8.4%。消费需求不断扩大，社会消费品零售总额达68.61亿元,是2001年的3.4倍，年均增长14%。

　　过去的五年，是精神文明和民主法制建设稳步推进的五年。广泛开展了群众性精神文明创建活动和社会主义荣辱观教育，全社会文明程度不断提高。认真执行市人大及其常委会的决议、决定，自觉接受人大的法律监督、工作监督和政协的民主监督，认真办理人大代表议案、建议和政协提案。加强同各民主党派、工商联、无党派人士和人民团体的联系，认真听取各界人士的意见、建议。村（居）民自治深入推进。政务公开不断扩大，政府决策科学化、民主化水平进一步提高。“四五”普法圆满完成，依法治市得到加强。创建“平安六盘水”工程深入推进，获社会治安综合治理全国先进地市称号。信访工作综合考核连续三年获全省第一。维稳工作成效明显。认真落实党风廉政建设责任制，加大案件查处力度，切实纠正行业不正之风，廉政建设不断深入。国防教育和国防后备力量建设得到加强。“双拥”创建活动扎实推进。民族、宗教、审计、监察、外事侨务、对台、人防、地方志等工作取得新的成绩。

　　各位代表：五年的奋力拼搏，增强了六盘水的实力，提升了六盘水的形象，扩大了六盘水的影响。成绩的取得，是省委、省政府正确领导，省直部门大力支持的结果；是全市各族人民团结奋斗、苦干实干的结果，是历届老领导、老同志打下的基础和长期关心的结果；是社会各界鼎力相助、积极参与的结果。在此，我代表市人民政府，向在过去五年中为六盘水市经济社会发展作出艰苦努力和付出辛勤劳动的全市广大工人、农民、知识分子、干部，向各民主党派、工商联、无党派人士和各人民团体，向驻市人民解放军、武警官兵、公安干警、民兵和预备役人员，向所有关心、支持和帮助我市改革开放和现代化建设的各界人士，表示衷心的感谢并致以崇高的敬意！

　　在取得成绩的同时，我们也清醒地认识到，欠发达、欠开发仍然是我市的基本市情，发展中还存在一些突出的困难和问题：农业基础脆弱、靠天吃饭的基本状况没有改变，农民增收困难，城乡差距还在拉大；经济增长方式粗放，资源环境对经济发展的约束性日益突出；生态脆弱，石漠化严重；交通、水利等基础设施还较薄弱；社会事业发展相对滞后；投资环境还有待改善，保持投资持续增长的机制还不健全，融资渠道狭窄，投资主体单一，社会投资不足；安全生产基础还不牢固，维护社会稳定的压力较大；政府职能、管理方式和工作作风的转变还不能完全适应经济社会发展的需要。对于这些问题，必须采取有力措施，认真加以解决。

对第六届市人民政府工作的建议

　　第六届市人民政府任期的五年，是我市全面完成“十一五”规划、加快建设小康社会、推进经济社会发展实现历史性跨越的关键时期。我们面临难得的发展机遇，主要是国家继续实施西部大开发和新阶段扶贫开发战略，鼓励东部地区产业向中西部地区转移，省委、省政府实施“煤―电―化”发展战略以及加快西部经济带建设。我们有着独特的优势，主要是区域位置和资源供给半径优势凸显，资源优势转化为经济优势的进程加快；改革开放以来特别是“十五”期间取得的巨大成就，产品产业结构的进一步优化，为我市新一轮发展奠定了坚实基础。我们要进一步增强信心，振奋精神，抓住机遇，发挥优势，把富民强市的宏伟大业推向前进！

　　今后五年，政府工作的总体要求是：以邓小平理论和“三个代表”重要思想为指导，全面贯彻落实科学发展观和构建社会主义和谐社会的重大战略思想，坚持能快则快、又好又快，紧紧围绕“三个基本”，大力实施工业强市、科教兴市、环境立市战略，加快工业化、推动城镇化、带动农业产业化，进一步改善经济社会发展的基础条件，进一步深化改革、扩大开放，着力推进经济结构调整，着力转变增长方式，着力促进社会进步，着力解决民生问题，加快推进经济社会发展的历史性跨越。

　　主要预期目标是：到2011年生产总值比2006年翻一番以上，年均增长15%以上；财政总收入比2006年翻一番以上，年均增长18%以上；五年累计完成固定资产投资900亿元左右；城镇居民人均可支配收入和农民人均纯收入年均分别增长8%以上；城镇登记失业率控制在4.2 %以内；人口自然增长率控制在7‰以内；单位生产总值能耗下降20%。

　　（一）加大“三农”工作力度，基本改变农村面貌。坚持“工业反哺农业、城市支持农村”和“多予、少取、放活”的方针，认真落实各项支农惠农政策，加大“三农”投入，引导社会各方面的资金、人才、技术、管理等生产要素向农业和农村流动，积极发展现代农业，扎实推进社会主义新农村建设。一是加强农村基础设施建设，大力发展农村公共事业，着力改善农村生产生活条件。实施农村安全饮水工程，解决55万人的饮水安全问题，使全市80%以上的农民喝上安全卫生水。加快实施户户通电工程，全面完成农村电网建设与改造。改造通村公路2500公里，实现三分之一以上的村通油路。加快发展农村广播电视事业，实现20户以上自然村寨通广播电视。完成全市农村茅草屋改造，解决农村无房户住房问题。加大沼气池建设力度，力争30%以上的农户稳定用上沼气。推进以村寨道路硬化、改厕改圈、排水排污、绿化美化为主要内容的村寨建设，使村容村貌得到较大改观。完善农村文化、卫生等设施，实现村村有文化活动室、村村有卫生室。二是加强农民培训，加快培养新型农民，着力提高农民整体素质。农村适龄儿童要一个不少地接受义务教育；加强对青壮年农民培训，平均每户至少一人掌握一门农业实用技术或非农劳动技能；采取学历培养、短期培训等方式，加强对乡土人才的培养，基本实现村村有1名合格的卫生员、畜牧兽医员和农民技术员；加大对村干部的培训，着力提高村干部的文化水平和办事能力，使村干部成为农村经济发展的明白人、带头人和组织者；广泛开展农民喜闻乐见和易于接受的健康有益的文化活动，采取示范引导等方式，逐步转变农民的思想观念和生产生活方式。三是抓好粮食生产、调整农业产业结构、加快富余劳动力转移，多渠道增加农民收入。加强基本农田建设，建成农业人口人均0.5亩高产稳产基本农田。高标准建成22万亩烟水配套工程。建成雨水集蓄利用工程30万立方米。新增、恢复和改善有效灌溉面积20万亩。推进农业科技进村入户，优良品种普及率和实用技术入户到位率达90%左右。稳步提高农业综合生产能力，粮食总产量达90万吨左右。大力调整农业产业结构，着力推进马铃薯、生态畜牧业、蔬菜、茶叶、经果林、中药材等优势特色产业的发展，建成一批上规模的农业产业化基地，培育一批上档次、产值上亿元的龙头企业，加快农业产业化进程。积极发展农民专业合作社，壮大农村经纪人队伍，完善公司、基地与农户的利益联接机制。加强农业技术推广体系、农村市场体系、农产品质量安全体系和动植物病虫害防控体系建设，强化农业产前、产中、产后服务。抓好农村劳动力转移培训，促进农村富余劳动力向城镇和二三产业有序转移40万人左右。抓好新阶段扶贫开发，解决农村贫困人口的温饱问题，实现低收入人口稳定脱贫。到2011年，全市农民人均纯收入达到3500元。

　　（二）大力推进产业结构调整，使产品和产业结构更加优化合理。做大做强支柱产业，发展相关产业，培育接续产业，拓宽发展空间，加速推进我市产业门类由单一向多元转变，由资源型城市向综合型城市转变，不断增强经济发展的持续能力。一是依托大企业，进一步做大做强煤炭、电力、钢铁、建材等传统支柱产业。做大原煤产量，力争原煤生产能力达8500万吨以上；扩大电力工业规模，力争电力装机容量达1000万千瓦左右；调整钢铁产品结构，大力发展板材、硬线、优型材等高附加值产品，提升钢铁产业实力；扩大水泥生产能力，积极发展新型墙体材料、装饰材料，促进建材产业上规模、上档次。二是依托传统支柱产业，延伸发展煤化工、高载能、矿山机电制造等相关产业。坚持把发展煤化工作为工业结构调整的主攻方向，着力推进“六大煤化工基地”建设，力争建成一批大型煤化工项目，促进煤炭产业从粗放向集约、产品从低端向高端转变，把煤化工发展成为重要产业之一；高起点发展电解铝、电解锌、电解黄磷等高载能产品及其精深加工；大力发展矿山机械和矿山机电制造业。三是加快发展轻工业。做大啤酒、农产品加工等食品工业，稳步发展制药工业，有序发展印刷包装、塑料制品等行业；以工业集中区为载体，大力发展加工制造业，积极承接东部地区产业转移，着力发展劳动密集型和技术密集型产业，促进加工制造业的规模化、集群化发展。四是大力发展现代服务业。培育壮大旅游业，大力开发乡村观光、休闲度假等旅游资源；加快发展现代物流业，建设贵州西部物流中心城市和南贵昆经济区重要的物流节点；大力发展金融保险、邮政通信、交通运输、商贸流通、餐饮娱乐等产业；积极发展文化、体育、卫生、广播电视、新闻出版等产业。五是创造条件，逐步发展高新技术产业。

　　（三）加强基础设施建设，基本形成适应发展需要的基础设施体系。树立“交通引领经济”理念，坚持交通建设优先。配合建成六沾铁路复线，完善六盘水铁路枢纽，建设一批专用铁路支线；以“一横一纵一环线”高速公路为主骨架，以二级公路为辅助干道，以三、四级经济路和通乡油路为配套线，加快推进公路网络建设，实现县县通高速公路或高等级公路、乡乡通油路。建成镇胜高速公路境内段和水盘高速公路，力争建成杭瑞高速公路境内段，推进市中心区绕城环线建设。到2011年，境内等级公路通车里程达4000公里以上；建成六盘水机场。以解决工程性缺水为重点，加强水利基础设施建设，基本实现城乡供水安全、饮水安全、防洪安全和水生态环境安全。重点促进双桥水库、白河沟水库、岱瓮水库等一批重要水库工程的建设，基本完成小（二）型以上病险水库除险加固；治理水土流失面积1200平方公里。加快“信息高速公路”建设，建立和完善覆盖城乡、高速灵活、安全可靠的邮政通信、宽带传输和用户接入网络，市、县、乡宽带接入网实现全覆盖，进村宽带接入网覆盖率达60%以上。加强城乡电网建设。

　　（四）加强资源环境工作，努力建设资源节约型和环境友好型社会。必须把节约资源作为根本要求，把保护环境作为发展底线，努力降低发展的资源和环境代价，提高经济发展的质量和效益，使有限的资源得到最大化、可持续利用，使六盘水的山更绿、水变清、空气更清新。大力发展循环经济，按照减量化、再利用、资源化的原则，推进节能节水节地节材，加强资源综合利用和回收利用，形成低投入、低消耗、低排放、高效益的节约型增长格局。严格执行行业准入标准和新上项目环保“三同时”制度，实施排污总量控制、排污许可证和环境影响评价制度，从源头上控制污染排放。加大对重点区域、重点行业和主要污染物的治理，大力实施三岔河和拖长江综合治理工程。到2011年，重点污染源废水、废气排放达标率90%以上，城镇集中式饮用水源地水质达标率95%以上，三岔河流域和拖长江流域河流出境断面水质按功能区达标率95%以上。全面完成国有重点煤矿采煤沉陷区综合治理。大力推进退耕还林、天保、珠防、石漠化综合治理等生态工程建设，全市森林覆盖率提高到35％，初步建成“两江”上游重要的生态屏障。

　　（五）大力实施城镇化战略，构建适应经济社会发展的城镇体系。加强城镇规划建设管理，加快推进城镇化进程，充分发挥城镇在区域经济发展中的带动作用，促进农村人口向城镇转移，引导各种要素向城镇聚集。把市中心区建设成为人口超过50万、城区面积达60平方公里以上的大城市；把红果、平寨建设成为具有较强辐射能力和较高综合服务水平的中等城市；加快发展一批有产业支撑、特色鲜明、适宜人居的城镇。到2011年，全市城镇化水平达40%以上。

　　加快市中心区基础设施和公用事业设施建设，进一步完善城市水、电、路、气等基础设施和教育、体育、文化、卫生等公用事业设施；加大城市绿化和治污力度，切实改善人居环境。建成环城林带。城市森林覆盖率达35%以上，城市绿地率达30%以上；城市污水处理率达60%以上，环境空气质量优良率达95%以上。大力实施水城河综合治理，确保两年内水变清、岸变美。加快“城中村”改造和旧城改造；坚持从学校、机关、社区、厂矿抓起，通过教育引导，大力提升市民素质；精心打造“凉都”品牌，加快把市中心区建设成为集消夏、度假、休闲、会展、高原体育运动训练为一体的特色城市。争创全国文明卫生城市。

　　（六）进一步深化改革、扩大开放，建立健全充满活力、富有效率、更加开放的体制机制。坚定不移地推进改革，破除影响发展的各种障碍，促进各类市场主体公平竞争。加快国有企业改革，完善法人治理结构，基本建立现代企业制度，国有资本基本退出竞争性领域；大力发展非公有制经济，形成多种经济成份共同发展、互为补充、相互交融、齐头并进的发展格局，到2011年，非公有制经济占生产总值的比重达50%以上；全面完成以“三改革一化解”为主要内容的农村综合改革；继续深化财政体制改革，基本建立公共财政体制框架；深化投融资体制改革，建立和完善新型投资体制，加快发展资本市场，鼓励和支持有条件的企业通过发行债券等方式扩大直接融资规模；加快文化、教育、公共卫生等社会事业改革，推进水利工程管理体制和集体林权制度改革；加快建立现代市场体系；进一步规范市场经济秩序；着力建设诚信社会。把改革与创新有机统一起来，创新体制机制，创新思路方法，重点鼓励和支持企业自主创新，努力建设创新型城市。坚定不移地扩大对外开放，大力实施“走出去”、“引进来”战略，提高对外开放水平。加强区域经济合作，加快发展对外贸易；改进招商引资方式，加大招商引资力度，力争招商引资实际到位资金年均增长25%。

　　（七）推进各项社会事业全面发展，增强经济社会发展的协调性。大力实施科教兴市和人才强市战略。坚持教育优先发展，促进教育公平，努力实现教育均衡协调发展。巩固提高九年制义务教育，确保全市适龄儿童入学率达99%以上、青壮年非文盲率达97%以上；大力发展普通高中和中等职业技术教育，努力实现初中毕业生升入普通高中和中等职业技术学校的人数各占50%；大力发展高等教育，建成1所本科院校。积极发展学前教育、成人教育、老年教育、特殊教育、民族教育、现代远程教育。强化师德建设，加强教师培训，着力提升教师素质。全面实施素质教育，提高教育质量。积极探索多元化投资办学机制，鼓励和支持发展民办教育。坚持重在培养和使用，加强人才队伍建设，促进人才总量增加、结构改善、素质提高，为经济社会发展提供智力支持和人才支撑。加大科技投入，加大科普力度，加快科技成果向现实生产力转化，着力提高全民科技素质和科技对经济增长的贡献率。

　　加快发展文化事业，满足群众文化需求，提升群众文化素养和文明素质。加强城乡公共文化服务体系建设，广泛开展群众性文化活动。挖掘、保护、发展民族民间文化。健全卫生服务、疾病预防和卫生执法监督体系，完善城乡基本卫生保健制度。整顿医疗卫生秩序，加强医德医风建设，提高医务人员业务水平，为群众提供安全、有效、方便、价廉的医疗卫生服务，着力解决群众看病难、看病贵问题。完成市人民医院改扩建工程，基本完成县级医院和乡镇卫生院改扩建工程。完善新型农村合作医疗制度，确保参合率达90％以上。大力开展全民爱国卫生运动，深入推进“整脏治乱”专项活动。加快发展广播电视事业，广播综合人口覆盖率达95%以上，电视综合人口覆盖率达98%以上，有线电视联通乡镇达80%以上。坚持控制人口数量与提高人口素质并重，进一步加强人口计生工作，建立和完善人口计生工作新机制，改善人口结构，引导人口分布，全市总人口控制在320万以内。

　　（八）着力解决人民群众最关心、最直接、最现实的利益问题，加快构建和谐社会。坚持把经济社会的发展与人民群众收入的增加、生活的改善和干部职工待遇的提高紧密结合起来，做到发展为了人民，发展成果由人民共享。强化就业再就业工作，重点做好城镇新增劳动力、农村富余劳动力、高校毕业生、退役军人和零就业家庭人员的就业，累计促进城乡就业40万人以上。全面建立社会保险、社会救助、社会福利、慈善事业相衔接的城乡社会保障体系。加快完善社会保险体系，健全企业职工养老保险制度，强化保险费征缴，逐步做实个人账户；深化机关事业单位养老保险制度改革；加快推进医疗、失业、工伤、生育保险制度建设。加强对困难群众的救助，完善城市低保，实现应保尽保；全面建立农村最低生活保障制度；加强救灾救济工作。大力发展以扶老、助残、救孤、济困为重点的社会福利，推进老龄事业和残疾人事业发展。大力发展慈善事业，切实开展各种形式的社会捐赠活动。牢固树立“以人为本、安全第一”的理念和安全发展观，加大安全生产工作力度，提高安全生产水平，切实维护群众生命财产安全。坚持以稳定保和谐，深入开展创建“平安六盘水”工程，全力维护社会稳定，确保人民安居乐业，社会安定有序。

　　（九）加强社会主义精神文明和民主法制建设，充分调动各方面的积极性。深入开展精神文明创建活动，树立以“八荣八耻”为主要内容的社会主义荣辱观，强化社会公德、职业道德和家庭美德教育，提高公民道德水准。倡导和谐理念，培育和谐精神，形成全社会共同的思想基础。坚持依法治市，加强民主法制建设。全面完成“五五”普法，着力提高公民法制意识和法律素质。扩大公民合法有序的政治参与，保证公民依法行使选举、决策、管理、监督等民主权利。各级政府要自觉接受人大及其常委会的法律监督和工作监督，自觉接受政协的民主监督。认真听取民主党派、工商联、无党派人士和人民团体的意见，接受新闻媒体监督。发挥工会、共青团、妇联等群众团体的桥梁纽带作用，完善社情民意的反映机制。落实民族宗教政策，加快民族地区经济社会发展。加强国防教育，推进民兵预备役队伍建设，深入开展“双拥”创建活动。继续发展妇女、儿童事业，进一步加强外事侨务、对台、档案、人防、气象、地方志等工作。

　　（十）加强政府自身建设，努力建设务实高效公正廉洁政府。一是切实转变政府职能，规范行政权力。推进行政管理体制改革，强化政府社会管理和公共服务的职能。进一步明确政府部门的权力和责任，减少行政审批事项，规范和优化审批程序。完善部门之间的分工协作机制，避免部门职能交叉和推诿扯皮。探索建立行政首长问责制。二是全面推进依法行政，规范行政行为。坚持有法必依、执法必严、违法必究，坚决克服多头执法、执法不公和执法违法等问题，强化行政执法监督和行政效能监察。坚持科学民主决策，健全重大决策集体研究、专家咨询、社会公示和听证等制度。三是大兴求真务实之风，狠抓工作落实。深入基层和实际，加强调查研究。切实把做好基础性工作作为长期坚持的执政理念，扎扎实实地推进发展。强化大局意识，做到令行禁止，确保政令畅通。大力整顿机关作风，提高服务质量和效率。加强学习，提高素质，适应新形势发展的需要。四是坚持执政为民，加强廉政建设。增进对群众的感情，关心群众疾苦，解决群众困难。建立健全教育、制度、监督并重的惩治和预防腐败体系，深入开展反腐败斗争，切实形成风清气正的政务环境。

2007年市人民政府工作的主要任务

　　今年政府工作的主要预期目标是：生产总值增长16%以上，财政总收入增长18%以上，全社会固定资产投资增长16%以上，全社会消费品零售总额增长12%以上，城镇居民人均可支配收入和农民人均纯收入分别增长8%以上，人口自然增长率控制在8‰以内，城镇登记失业率控制在4.3%以内，单位生产总值能耗降低4%左右，主要污染物排放减少2%。

　　围绕实现以上目标，重点做好以下工作：

　　（一）抓好“三农”工作。加大以中低产田土改造为主的基本农田建设力度，加强以水、电、路、气为重点的农村基础设施建设。实施好雨水集蓄利用工程；高标准建设烟水配套工程，完成灌溉面积2.29万亩；继续实施农村饮水安全工程，解决5万人饮水安全问题；加快农村电网改造；完成129个20户以上自然村寨农村广播电视“村村通”工程；改造通村公路1000公里；建成沼气池1.85万口。多形式开展农民培训，重点加大对青壮年农民的农业实用技术和非农劳动技能培训。完成“阳光工程”培训3.6万人，其中职业技能示范性培训0.9万人，引导性培训2.7万人；大力促进农村富余劳动力转移，完成劳务输出6万人，其中有组织劳务输出0.45万人；继续实施村级卫生员和畜牧兽医员培养计划；进一步加强对乡土人才的培训和培养。推进农业产业结构调整，继续发展粮食生产；稳定发展烤烟、油菜等经济作物；做大马铃薯产业；加快发展畜牧业，抓好盘县、水城县种草养畜试点工作，力争畜牧业增加值增长10%以上。加大对农业产业化基地的扶持，培育和壮大龙头企业，鼓励农技人员采取领办公司等形式发展农业产业化。继续推进新阶段扶贫开发，实施整村推进计划，逐户落实帮扶措施，完善扶贫办法，改革投入方式，建立脱贫长效机制，净减少农村贫困人口和低收入人口各0.92万人。加强农村基层组织建设，全面完成村支书（主任）培训任务。

　　（二）加快发展工业经济。抓好响水煤矿、发耳煤矿、玉舍煤矿东中西井、松河煤矿等骨干矿井建设，推进现有煤矿整合改造，做大做强地方煤矿，力争原煤产量达5000万吨；加快发展煤化工，一季度开工建设老鹰山煤基气化替代燃料项目一期工程，年内争取核准建设柏果煤化工基地项目，继续做好其他煤化工基地项目的前期工作；大力支持建成盘南电厂二期工程，尽快完成发耳电厂核准并建成一期工程，推进盘县电厂技改扩容，力争六枝电厂尽快立项，继续做好其他后续电源项目的前期工作；支持水钢搞好技改扩能，大力调整产品结构，加快中厚板轧制生产线建设；根据市场需求和地缘条件，合理布局并扩大水泥生产能力，力争木岗、老鹰山、红果等地的水泥项目落地；积极利用煤矸石、粉煤灰等主要工业废弃物发展新型墙体材料、装饰材料；大力发展高载能产业，支持双牌铝业公司加快技改工程及铝加工项目建设；支持水矿集团博力公司支架厂投产，支持六盘水煤机厂通过改革改制做大做强；支持重庆啤酒集团六盘水有限责任公司搞好技改，做大啤酒产量。支持市食品总厂通过改制做大做强。以建设工业集中区为载体，通过招商引资，加快引进建设劳动密集型、技术密集型产品、产业和产业关联度高、辐射带动能力强的企业。红山、石桥两个工业集中区的建设要取得重大突破，其中红山工业集中区要力争实现水、电、路“三通”，并争取一批企业进入；石桥工业集中区要在完成规划的基础上，力争开工建设水、电、路等基础设施。积极规划建设六枝特区和盘县的工业集中区。

　　（三）加大固定资产投资力度。坚持扩大固定资产投资规模不动摇，坚持抓项目建设不放松，改善投资结构，提高投资效益。一是积极发展资本市场。推进多种所有制金融机构的建立，引导民间资金进入资本市场。加强中小企业信用担保体系建设，建立健全担保机构的风险补偿机制。充分发挥投资公司在投融资方面的重要作用。积极支持有条件的企业通过发行债券等方式，扩大直接融资规模。加强金融生态环境建设，引导金融机构加大对各个发展领域、各种投资主体的支持力度。完成六盘水市商业银行的组建。继续支持农村信用社改革发展，充分发挥政策性金融的作用。大力推广盘县小额扶贫信贷经验，着力解决农村金融缺失问题。二是努力争取国家和省在政策、项目、资金上加大对我市的支持力度。三是依托大企业、大项目，以产权多元化和投资主体多元化拓宽投融资渠道，扩大投融资规模。四是继续加大财政支出结构调整力度，充分发挥政府对投资的引导作用，运用补助、贴息等扶持政策，促进各方面的资金投向自主创新和产业升级、资源节约和环境保护等领域。做深、做细、做实项目前期工作，整合项目库，建立健全项目滚动推出机制。加强项目建设管理。做好项目落地的各项服务工作，确保项目顺利实施。

　　（四）加强交通水利基础设施建设。配合做好六沾铁路复线及配套项目建设，开工建设水盘高速公路，加快柏火二级公路改造，推进红威公路、玉马公路、英柏公路等二级公路建设，建设通乡油路150公里；完成县乡公路管理体制改革，加大乡村公路管护力度。继续抓好六盘水机场的前期工作。做好白河沟水库开工建设的准备和双桥水库前期论证工作，完成许家屯、长海子、纳麻、泥着落等病险水库除险加固工程。

　　（五）加强城镇规划建设管理。完成市中心区人民路改造和东西延伸段建设，建成凉都大道，开工建设乌蒙大道，完成市中心区36条16.7公里小街小巷的综合改造。启动实施水城河综合治理工程，建成水城河污水收集管网系统，正式运行污水处理厂，力争开工建设市中心区防洪治涝二期工程。完成10万立方米城市燃气储气柜扩建工程。开工建设凉都体育中心。进一步完善市中心区垃圾处理系统，开工建设医疗垃圾处置中心。启动建设六盘水粮油产品流通中心。完善市中心区农贸市场基础设施，建成3个农产品检测检验中心。树立“现代城管”理念，大力推进公众城管、精细城管、科学城管。加强社区建设，规范物业管理。进一步理顺城市公交管理体制，实现城市公交规范运营、健康发展。办好第四届“凉都消夏文化节”，不断扩大“中国凉都”的知名度和影响力。继续推进县城和小城镇建设。

　　（六）切实做好资源环境工作。一是大力发展循环经济。积极争取把我市列入国家和省循环经济试点。二是大力推进节能降耗。以高耗能行业和年耗能5000吨标准煤以上企业的节能降耗工作为重点，加快节能降耗技术改造。把分解到县、区和重点企业的节能降耗目标落实到位，确保完成。三是合理利用和保护资源。完成市级土地利用总体规划修编纲要的审批。严格保护耕地特别是基本农田。加强矿产资源的勘查、保护和开发管理，强化对矿山企业开采回采率、采矿贫化率、选矿回收率的监管。继续整顿和规范矿产资源开发秩序，积极推行煤炭资源有偿使用制度，启动矿权储备交易工作。大力开展节水活动，严格控制抽采地下水。四是加强环境保护。大力开展“环境保护年”活动，建立督查制度和定期公布排污指标制度。加大环保执法力度，依法淘汰一批污染严重的企业，着力改造一批环保设施欠缺的企业，在线监管一批污染负荷高的企业，限期治理一批排污不达标的企业。采取有力措施，坚决防止“两土”反弹，按计划关停不符合国家产业政策的小机焦和小洗选。启动国有重点煤矿采煤沉陷区综合治理工程。实行矿山地质环境治理恢复保证金制度，加强地质灾害防治。五是继续推进生态建设。完成营造林面积10万亩。大力争取国家将我市列入石漠化综合治理试点。抓好六枝特区集体林权制度改革试点工作。

　　（七）大力发展非公有制经济。一是加大支持力度。坚持公平公正和“非禁即允”的原则，鼓励和支持非公有资本以各种方式进入法律和政策没有限制的行业和领域，鼓励和支持非公有制经济参与国有企业改革改制。二是加大引导力度。加强对非公有制企业的政策扶持、技术扶持和项目引导，改善其投资结构、提高其投资效益，促进非公有制经济发展壮大；引导非公有制企业完善法人治理结构，促进其管理上台阶；引导非公有制企业增强社会责任感，积极参与公益事业和慈善事业。三是加大服务力度。简化办事程序、提高服务质量，坚决制止吃拿卡要，为非公有制经济营造良好的发展环境。加强信息服务，及时、充分地向社会公布政府的产业布局和政策信息。强化技术和人才服务，提升非公有制企业素质。

　　（八）进一步深化改革、扩大开放。全面启动以“三改革一化解”为主要内容的农村综合改革。加快以“三改一加强”为重点的国有企业改革，完成30%以上国有商贸企业改革。对于涉及公共服务领域和事关国计民生的企业改革，必须坚持由政府控股。深化部门预算和国库集中支付改革，完善政府采购制度，逐步健全公共财政体制。强化预算约束，加强财政资金使用监管，提高资金使用效率和效益。完成行政事业单位国有资产的清查并加强监管。继续整顿和规范市场经济秩序，严厉打击非法传销、欺行霸市、价格垄断等行为。

　　加快建立改善投资环境的长效机制，加大力度整治投资软环境，通过良好的环境吸引市外资金参与我市发展。坚决避免靠低价出让资源开发权、降低能源价格和劳动力工资来吸引客商的不可持续做法。把招商与选商结合起来，以珠江三角洲、长江三角洲为招商引资的重点地区，大力引进一批农业产业化龙头企业、加工制造业企业、大型工矿企业，严禁引进高消耗、高污染、低水平的项目。加快人员、产品、产业“走出去”的步伐，提升六盘水经济的辐射和影响力。积极参与“泛珠三角”等区域经济合作，继续加强与对口帮扶城市、友好城市的经济技术合作和友好往来。

　　（九）大力发展各项社会事业。进一步巩固“两基”攻坚成果，迎接国家督导检查验收。完成30所农村义务教育阶段薄弱学校改造工程，建成市中心区范围内钟山区4所中小学、水城县双水2所中小学并实现秋季招生。大力发展普通高中教育，加快市三中凤凰校区、市一中双水校区建设进度。加快发展以就业为导向、以中等职业教育为重点的职业教育，完成中等职业教育招生1.2万人。继续推进高等教育加快发展。开工建设市人民医院综合住院大楼，建成20个乡镇卫生院，改造完善一批村级卫生室，进一步提高新型农村合作医疗参合率。加快信息化建设，全面实施全市有线电视网络资源整合，做好数字电视平移的准备工作。突出基础工作和基层队伍建设两个重点，切实抓好人口计生工作。继续实施“少生快富”工程，推进“关爱女孩行动”，综合治理出生人口性别比失衡问题，继续实施出生缺陷干预工程，切实加强对流动人口的服务和管理。

　　（十）扎实抓好安全生产工作。正确处理好安全与生产的关系，真正做到不安全不生产、抓安全促生产，确保安全生产指标控制在省下达的范围内。落实预防为主、综合治理的工作措施，加强对从业人员、管理人员、监管人员的培训，提高人员队伍素质。多渠道增加安全生产投入，不断提高安全装备水平和改善安全基础设施，重点要督促煤矿企业依法建立煤矿强制性安全投入制度，加强对煤矿维简费、安全生产费用使用情况的监督检查，督促指导煤矿推进安全质量标准化建设，打牢安全生产基础。理顺应急救援指挥体系，整合救护资源，提高灾害和事故的救援水平。建成六盘水市安全生产信息网。严格执行安全生产责任制，强化领导责任、监管责任、主体责任、岗位责任的落实。健全安全生产监管体系，加强安全监管，切实消除各种安全隐患。按照“四不放过”原则，严肃追究安全事故责任，坚决执行安全生产“一票否决”。

　　（十一）积极构建和谐社会。一是加强就业再就业工作。继续推进新一轮就业政策的落实，强化就业培训，提高劳动者就业技能，转变就业观念，搞好就业指导，千方百计扩大就业，促进城乡就业7.9万人，其中城镇新增就业1.9万人，下岗再就业0.1万人。二是加快完善社会保障体系。规范和强化城镇居民最低生活保障工作，积极推进农村特困救助制度向农村最低生活保障制度过渡。继续扩大城镇职工基本养老、医疗和失业、工伤、生育等保险覆盖面；加强社会保险基金监管，确保基金的安全与完整。切实做好对困难群体在医疗、教育、住房等方面的救助，加强对城镇生活无着的流浪乞讨人员的救助，认真落实农村“五保”供养政策。三是大力推进创建“平安六盘水”工程，全力维护社会稳定。加强社会治安综合治理，建立和完善群防群治的社会治安防控体系。启动全市社会治安技术防范控制系统建设。依法严厉打击黑恶势力犯罪、“两抢一盗”、多发性侵财犯罪等刑事犯罪；大力开展以废旧物资回收、二手市场交易等为重点的专项整治。进一步加大禁毒工作力度，充分发挥人民群众在禁毒工作中的主体作用，切实打好禁毒人民战争，遏制毒品问题发展蔓延势头。加快建立健全应急机制，预防和妥善处置各种群体性事件，提高保障公共安全和处置突发事件的能力。认真做好人民来信来访工作，及时化解各种社会矛盾。高度重视并积极解决土地承包、土地征用、房屋拆迁、环境污染、企业改制、安全生产、食品药品安全、涉法诉讼以及建设领域拖欠工程款和农民工工资等方面群众反映强烈的问题。

　　各位代表：回首过去，我们豪情满怀；展望未来，我们信心百倍！加快发展、实现跨越，是时代的要求，是人民的重托，是历史赋予我们的神圣使命。让我们高举邓小平理论和“三个代表”重要思想的伟大旗帜，紧密团结在以胡锦涛同志为总书记的党中央周围，全面贯彻落实科学发展观，在省委、省政府和市委的领导下，团结和依靠全市各族人民，励精图治，开拓进取，为实现富民强市的宏伟目标，为六盘水更加美好的明天而努力奋斗！
